# Adaptive response of Dongzhaigang mangrove in China to future sea level rise

**DOI:** 10.1038/s41598-022-15774-7

**Published:** 2022-07-07

**Authors:** Rongshuo Cai, Ruyi Ding, Xiuhua Yan, Cuihua Li, Jiang Sun, Hongjian Tan, Wu Men, Haixia Guo, Cui Wang

**Affiliations:** 1 Third Institute of Oceanography, Ministry of Natural Resources, Xiamen, 361005 China; 2 Institute of Marine Sciences, Shantou University, Shantou, 515063 China; 3 School of Marine Sciences, Nanjing University of Information Science and Technology, Nanjing, 210044 China

**Keywords:** Ecology, Environmental sciences, Natural hazards, Ocean sciences

## Abstract

Mangrove forests are valuable intertidal ecosystems that provide coastline protection, biodiversity maintenance, and carbon sequestration. However, their survival is under severe threat from rapidly rising sea levels. In this study, we aimed to investigate the changes in the area of the Dongzhaigang mangrove in China since the 1950s and causes of these changes using literature and remote sensing data. The impact of historical and future sea level rise (SLR) on the mangroves was analyzed using remote sensing data and climate model data under the low, intermediate, and very high greenhouse gas emission scenarios (Representative Concentration Pathways (RCPs) 2.6, 4.5, and 8.5). The area of the mangrove forests decreased from 3416 to 1711 hm^2^ during 1956–1988 and remained constant at 1711 hm^2^ after the 1990s, owing to anthropogenic disturbances such as reclamation and aquaculture before the 1980s and the protection of nature reserve establishment after the 1990s, respectively. Under RCPs 4.5 and 8.5, SLR is expected to cause > 26% of the mangroves to disappear by 2100, whereas under RCP 2.6, only 17% of the mangroves will likely be lost. Biological measures such as reestablishment of ponds as mangrove forests, afforestation, and biological embankment for sediment trapping in coastal wetlands are recommended to enhance the resilience of mangroves to SLR.

## Introduction

Mangroves are woody biomes composed of trees and shrubs in the tropical and subtropical coastal intertidal wetlands, with important multiple ecosystem functions including coastal protection, climate regulation, carbon sequestration, and food services^1–4^. They play an important role in the adaptation to and mitigation of the impacts of climate change and in social sustainable development in coastal areas. However, mangroves are vulnerable to rising sea levels and extreme events such as strong typhoons and droughts related to global warming^5–8^. If the vertical soil surface accretion rates of mangrove wetlands are not equal to or smaller than that of sea level rise (SLR), in other words, if the mangrove wetlands cannot adjust the soil surface elevation through sediment accumulation accretion, they cannot adapt to SLR and become at risk of disappearance^9–11^. Mangroves will very likely struggle to adapt and survive, if the rate of global SLR reaches 6.1 mm year^−1^ in the next 30 year^7^. Under the very high greenhouse gas (GHG) emission scenario (Representative Concentration Pathway (RCP) 8.5), only localized sedimentation accretion effects in mangrove wetlands can adapt to rising sea levels by 2055 and 2070^12^. In the Indo-Pacific tropics, the current rate of SLR exceeds the vertical accretion rate of the mangrove wetland surface at 69% of all sites studied. In areas with low tidal ranges and low sediment supplies, mangroves may be inundated as early as 2070^13^. In the Caribbean mangrove margin, mangrove growth rates are equal to the rate of SLR. However, if this rate exceeds 5 mm year^−1^, the mangrove islands in the Caribbean are unlikely to persist under the impact of SLR^14^. The evaluation of the characteristics and responses of mangroves to the effects of SLR can provide a scientific basis for formulating adaptation measures for mangrove systems and for the sustainable development of the economy, society, and environment in coastal areas.

**Figure 1:**
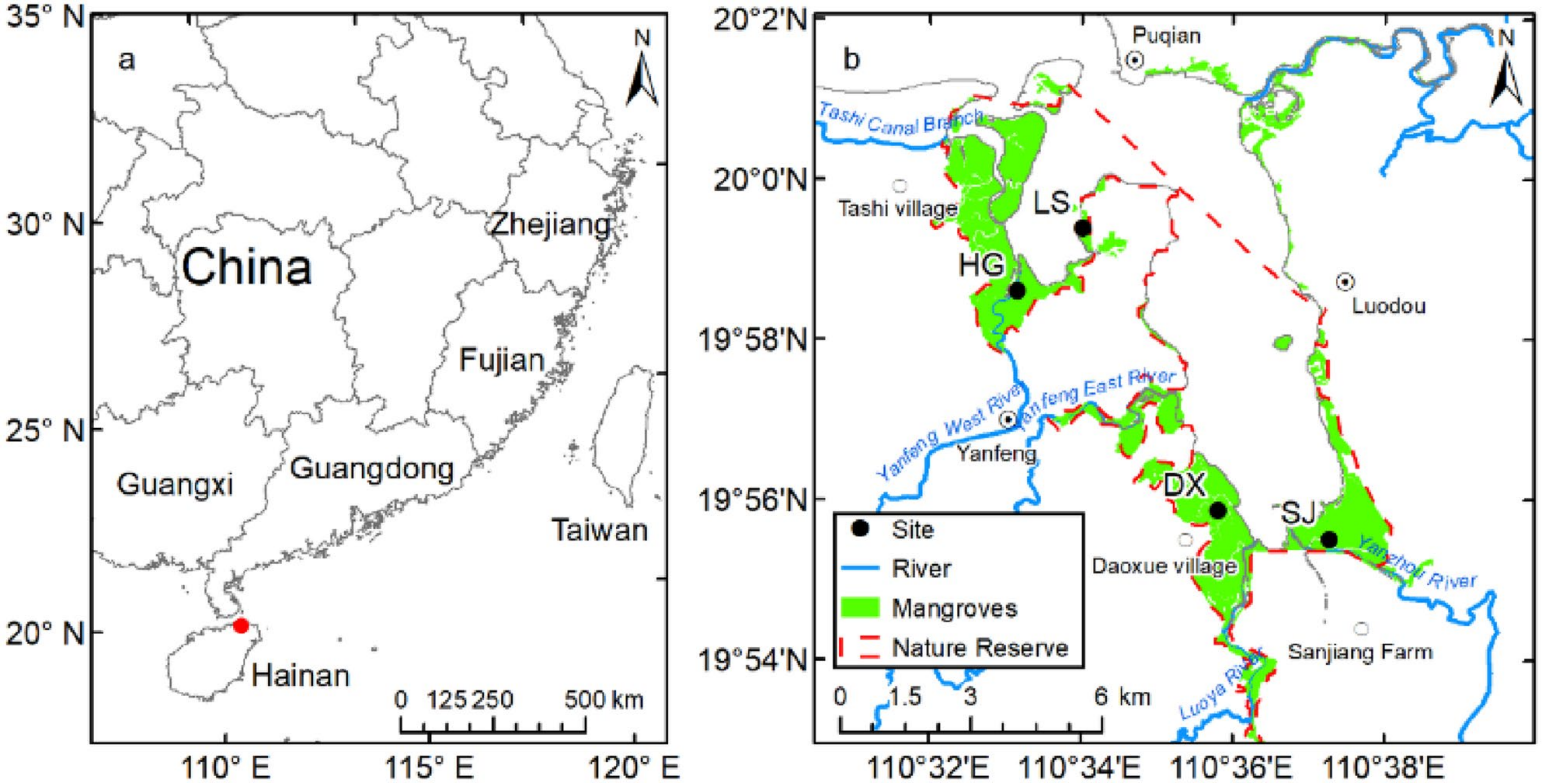
(**a**) Geographical location and (**b**) spatial distribution of mangroves around Dongzhaigang, Haikou City, Hainan Province, China. LS, HG, DX, and SJ refer to Linshi Village, Hegang Village, Daoxue Village, and the Sanjiang farm, respectively. (Partial data from: Zhang et al.^21^). Maps generated in ArcMap v10.0 (https://www.esri.com/en-us/home).

In China, mangroves are mainly distributed in the tropical and subtropical coastal areas of Hainan, Guangxi, Guangdong, Fujian, and Taiwan provinces, covering an area of approximately 21,148–24,801 hm^2^^15^ (Fig. 1a). The largest contiguous mangrove area and richest mangrove species are in the Dongzhaigang National Nature Reserve in Haikou City, with a small portion distributed in Wenchang City, Hainan Province (hereinafter referred to as “Dongzhaigang mangrove,” Fig. 1b). This area is the earliest established national mangrove reserve in China (established in 1980), with a total area of 3337.6 hm^2^ (within the red dashed line, Fig. 1b), accounting for 97% of the mangrove plants that comprise 19 families and 35 mangrove plant species in China^16^. The mangrove forests in and around the protected area are mainly distributed in four regions: Tashi, Yanfeng, Daoxue, and Sanjiang (Fig. 1b). From the 1960s to the 1980s, the area of Dongzhaigang mangrove decreased from 3416^17^ to 1600 hm^2^^18,19^ (in the range of 1575–1812 hm^2^) owing to human activities such as reclamation and land use^17,19,20^. Since the 1980s, Dongzhaigang mangrove has been protected, and its area has remained stable because of the establishment of provincial and national nature reserves.

Global warming has increased the rate of coastal relative SLR (RSLR) in China in recent decades (3.4 mm year^−1^, 1980–2020) compared to the global average^22,23^. The RSLR in the Dongzhaigang area was 4.6 mm year^−1^ from 1980 to 2018, which is much higher than the average value for coastal China. As the RSLR is expected to accelerate in the future^24,25^, the impact of rapidly rising sea levels on the mangroves in Dongzhaigang and other areas will become increasingly apparent and severe. Here, a new question has thus emerged: Can Dongzhaigang mangrove adapt to the effects of rapidly rising sea levels^9,23^ if its area remains unchanged and human activity does not increase? It is currently unknown what the related countermeasures for Dongzhaigang mangrove are required for adaptation to rapidly rising SLR. To date, few quantitative studies have been conducted in this area, although the threat to mangroves from SLR is real around the world^6,11,26^.

The main objective of this study was to analyze the changes in the area of Dongzhaigang mangrove over the past 60 years and the reasons for these changes using the results of previous work and remote sensing data. Since spatial representation is somewhat lacking, a supplementary investigation of sediment vertical accretion rates in mangrove wetlands in Hegang Village in Yanfeng and Sanjiang farm in Sanjiang (Fig. 1b) was implemented, although historical data of Dongzhaigang mangrove wetlands at sites such as Linshi and Daoxue villages were available^27,28^. Then, based on the analysis of the historical and future relative sea level changes in Haikou City, where Dongzhaigang is located, the effects of SLR on mangroves in Dongzhaigang under low, intermediate, very high GHG emission scenarios, RCPs 2.6, 4.5, and 8.5, were determined. Finally, the measures required to adapt to rising sea levels in Dongzhaigang mangrove were discussed. The results of this study will provide important insights into the mangrove conservation and management efforts in China.

## Results

### Historical changes and current status of the Dongzhaigang mangrove area

**Figure 2:**
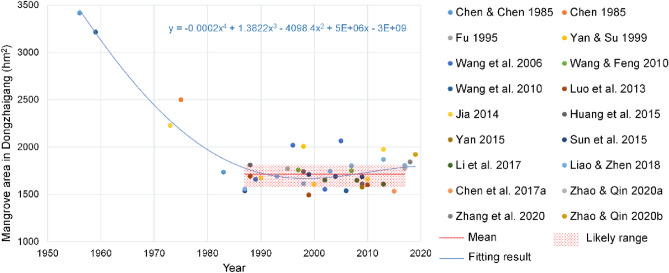
Changes in the mangrove area in Dongzhaigang from 1956 to 2019. The equation in the upper-right-hand corner of the plot refers to the fitting equation of historical changes in the total area of Dongzhaigang mangrove.

**Figure 3:**
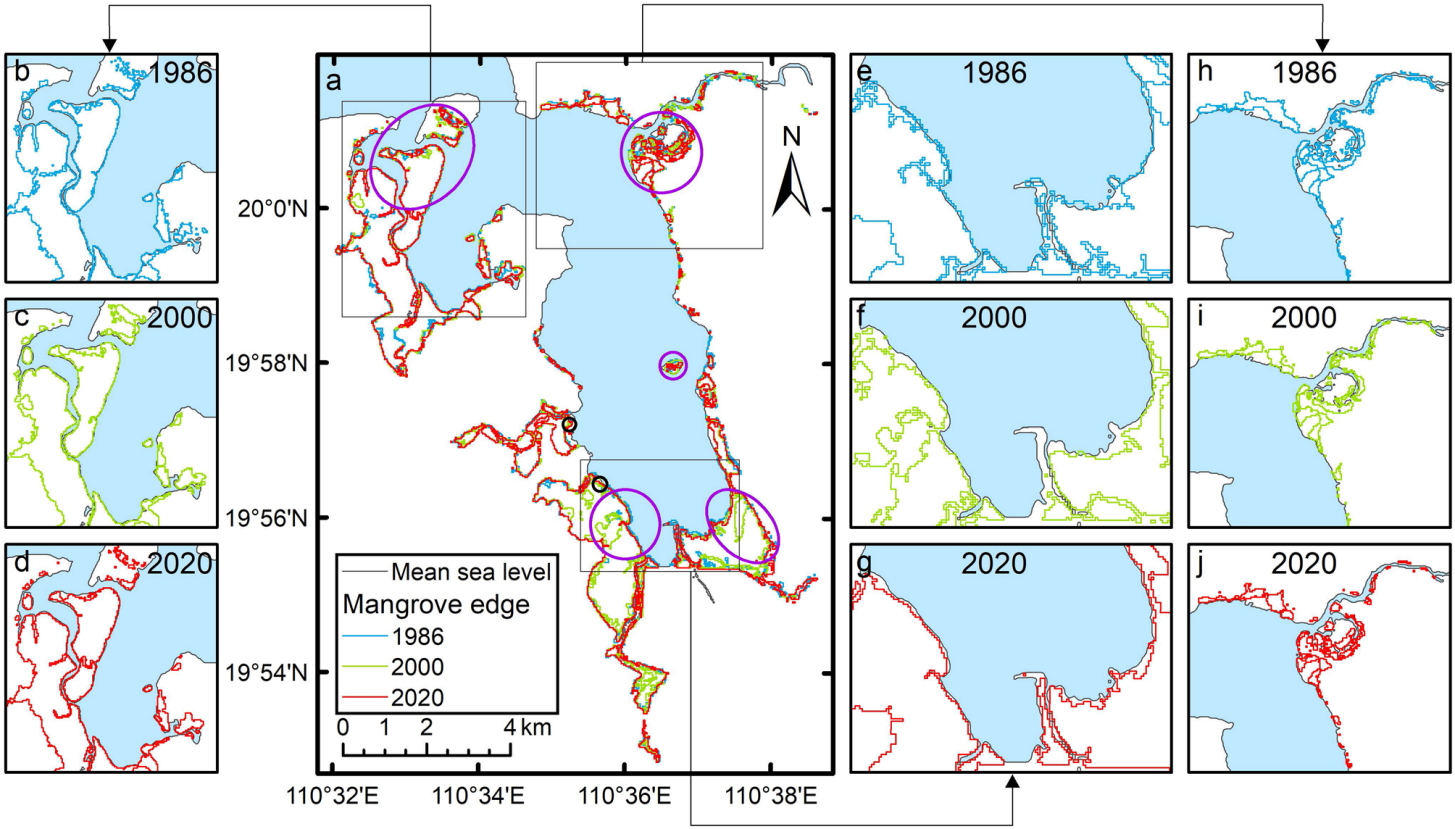
The dynamic changes in low mangrove edges in Dongzhaigang from 1986 to 2020. Maps generated in ArcMap v10.0 (https://www.esri.com/en-us/home).

Based on the literature and remote sensing data, we calculated the changes in the area of mangrove forests in Dongzhaigang since the 1950s presented in Fig. 2. In the last 60 years, the area of mangrove forests in Dongzhaigang has experienced large fluctuations mainly due to human destruction and protection activities such as mariculture reclamation, cofferdams, and restoration: it decreased from 3416 hm^2^ in 1956^17^ to 3213 hm^2^ in 1959^19,29^ and then decreased sharply to 1733 hm^2^ in 1983 and to 1537 hm^2^ in 1987^20,30^. Since the establishment of the national nature reserve in 1986, the decline in area of Dongzhaigang mangrove has stopped^19^, which are now protected and restored owing to the law and regulations that prohibit human activities from destroying the mangrove resource. In 1988, the area was restored to 1809 hm^2^, and since the 1990s, it has no longer decreased, remaining constant at approximately 1711 hm^2^ (in the range of 1575–1812 hm^2^) based on the literature)^18,20,31–34^ (Fig. 2). The area of the Dongzhaigang mangrove forest in 2019 was estimated to be 1842 hm^2^ based on the latest 2 m resolution remote sensing data^21^. Hence, we wonder how SLR has impacted Dongzhaigang mangrove in the past decades. However, it is very difficult to analyze how SLR has historically impacted the spatial changes in the Dongzhaigang mangrove; the same can be said regarding the influence of human activities, such as destruction before mid-1980s and protection after 1990s. However, the dynamic changes among low plant edges in the intertidal zone can be used to analyze the impact of natural driving forces such as SLR^35^, based on the latest remote sensing data for the period of 1986–2020. Thus, we analyzed the dynamic changes in low mangrove edges (hereafter, the edges), which are mainly impacted by natural impact drivers, as shown in Fig. 3. The dynamic low mangrove edges represented by 1986, 2000, and 2020 reveal the changes in spatial distribution of Dongzhaigang mangrove. As shown in Fig. 3. Most of the edges along the coast of Dongzhaigang between 1986 and 2020 migrated landward, but not significantly. However, if we look at the changes in detail, some edges such as those in Daoxue, Sanjiang (purple circles in Fig. 3a) more clearly retreated landward compared to other places. Besides, some edges of Luodou along the northeastern coast of Dongzhaigang outside the reserve and an unnamed small island (purple circles in Fig. 3a) also migrated landward very distinctly. On the contrary, the two smaller shore lines (black circles) in the northern part of Yangfeng and Daxue districts showed seaward expansion (Fig. 3a).

### Vertical rate of sediment accretion in mangrove wetlands

**Figure 4:**
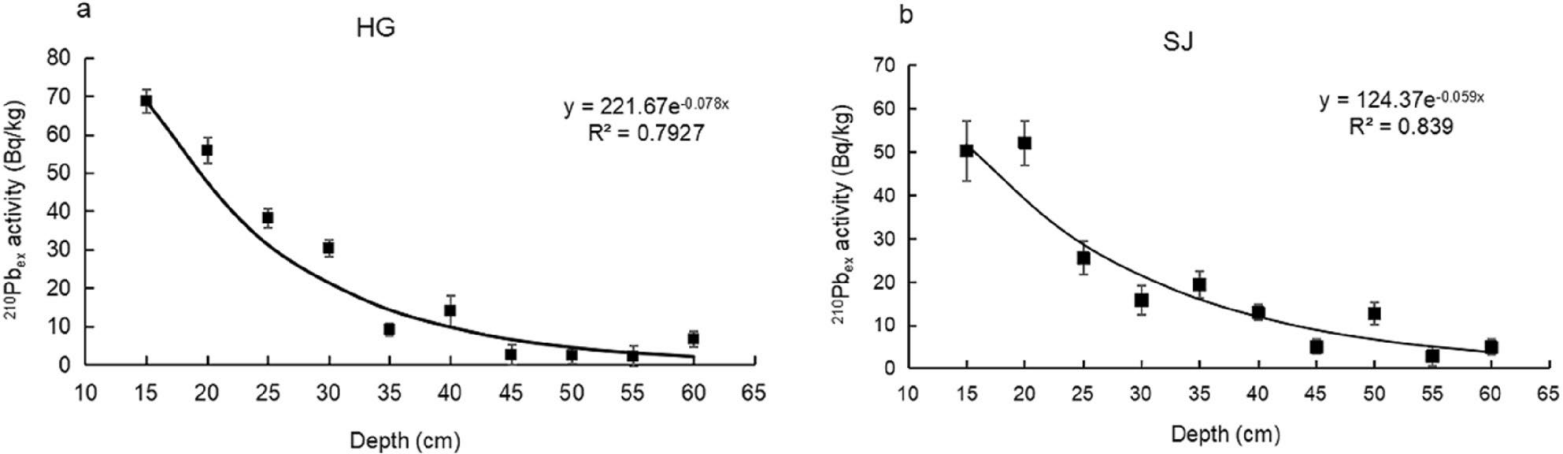
^210^Pb_ex_ activity profiles in selected cores such as from (**a**) station HG and (**b**) station SJ.

The vertical rate of sediment accumulation in mangrove wetlands can reflect whether the mangroves can adjust the soil surface elevation change through sediment trapping to adapt to SLR^6,11^. The vertical sediment accretion rates at two sites of Dongzhaigang mangrove (i.e., Linshi and Daoxue villages in Fig. 1b) can be obtained from historical documents, which are 0.41 cm year^−1^ at LS and 0.64 cm year^−1^ at DX, respectively^27,28^. Since historical data may not be enough to reflect the vertical sediment accretion rates in time and space, we conducted a supplementary investigation on the sediment accumulation rates at site HG in Yanfeng and SJ site in Sanjiang farms, respectively (Fig. 1b), based on the assumption that they can reflect the sediment supplies from main reivers such as Yanfeng West River and Yanzhou River, respectively. Sediment accretion rates measured using ^210^Pb_ex_ specific activity in the cores from sites HG and SJ showed that ^210^Pb_ex_ decayed exponentially with increasing depth, and the R^2^ values of both cores were approximately 0.80 after curve fitting. This analysis resulted in vertical sediment accretion rates of 0.53 and 0.40 cm year^−1^ at HG and SJ, respectively (Fig. 4). Therefore, the locations of sediment cores at sites LS, DX, HG, and SJ can basically represent the whole Dongzhaigang mangrove forest area.

### Rate of relative sea level rise in Dongzhaigang mangrove

**Figure 5:**
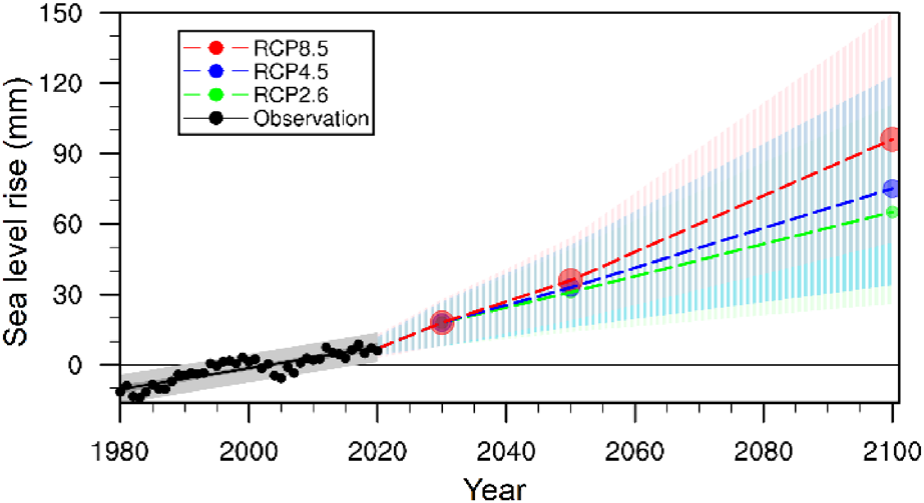
Historical and future relative sea level changes along coastal Dongzhaigang, Haikou City from 1980 to 2100; the 5–95% uncertainty ranges are shaded for RCPs 2.6, 4.5, and 8.5, respectively.

The global mean sea level (GMSL) is accelerating due to global warming-induced thermal expansion of the oceans and melting of land-based glaciers and ice caps into the sea^36^. Between 1901 and 2010, the GMSL rose by 0.19 m^9^. Coastal China is among the regions that experience the highest levels of SLR^23^. The rate of RSLR along China’s coast from 1980 to 2019 was 3.4 mm year^−1^, higher than the global average^23^. In the future, under the premise of increasing anthropogenic GHG emissions, global sea levels will rise rapidly, and it is projected that the GMSL may rise by 0.84 m (0.61–1.10 m) relative to the current levels by the end of the twenty-first century^9^. Based on the observations from the tide gauge stations in the Haikou area and model data from the Coupled Model Intercomparison Projection 5 (CMIP5), the rate of RSLR around Dongzhaigang reached 4.6 mm year^−1^ from 1980 to 2018. This rate is much higher than the global and China’s average values^23,25^ and will likely accelerate further in the future. Based on the results of the CMIP5 model simulations under different GHG emission scenarios^24^, the RSLR in coastal Haikou waters, including in Dongzhaigang, is expected to be significant by 2030, 2050, and 2100 for the low, intermediate, and very high GHG emission scenarios RCPs 2.6, 4.5, and 8.5, respectively (Table 1, Fig. 5). Under RCPs 2.6, 4.5, and 8.5, the sea level will rise by 65 (42–90, likely range), 75 (51–102, likely range), and 96 (70–125, likely range) cm by 2100, respectively, with the average RSLR rates of 6.84 (4.42–9.47, likely range), 7.89 (5.37–10.74, likely range), and 10.1 (7.37–13.12, likely range) mm year^−1^, respectively.

**Table 1:** Estimated coastal relative sea level rise (cm) and its rate (mm year^−1^) in the Haikou area under different GHG emission scenarios (data from Kopp et al*.*^24^).

Year	RCP 2.6			RCP 4.5			RCP 8.5		
	Mean	17–83% (likely)	5–95% (very likely)	Mean	17–83% (likely)	5–95% (very likely)	Mean	17–83% (likely)	5–95% (very likely)
**Relative sea level rise (cm)**									
2030	18	12–23	8–27	18	12–23	8–27	18	12–24	8–28
2050	31	21–42	14–49	33	23–43	16–51	36	26–46	19–54
2100	65	42–90	26–111	75	51–102	34–123	96	70–125	52–151
**Rate of relative sea level rise (mm year**^**−1**^**)**									
2030	7.2	4.8–9.2	3.2–11.08	7.2	4.8–9.2	3.2–11.08	7.2	4.8–9.6	3.2–11.2
2050	6.89	4.67–9.33	3.11–11.09	7.33	5.11–9.56	3.56–11.33	8.0	5.78–10.22	4.22–10.2
2100	6.84	4.42–9.47	2.74–11.68	7.89	5.37–10.74	3.58–12.95	10.1	7.37–13.12	5.47–15.9

Local settling rate: 1.09 ± 3.22 mm year^−1^.

### Impact of relative sea level rise on Dongzhaigang mangrove

Mangroves cannot easily adapt to rising sea levels if the rate of GMSL rise exceeds 6.1 mm year^−1^ (> 90% probability, very likely), whereas the survival threshold for mangroves is extremely likely to be exceeded (> 95% probability, extremely likely) when the rate of GMSL exceeds 7.6 mm year^−1^^7^. Although these values are based on global levels^7^, they still reflect the threat of SLR to local mangroves. In view of this, we further analyzed the potential impact and risks to Dongzhaigang mangrove from future SLR under different climate scenarios.

**Figure 6:**
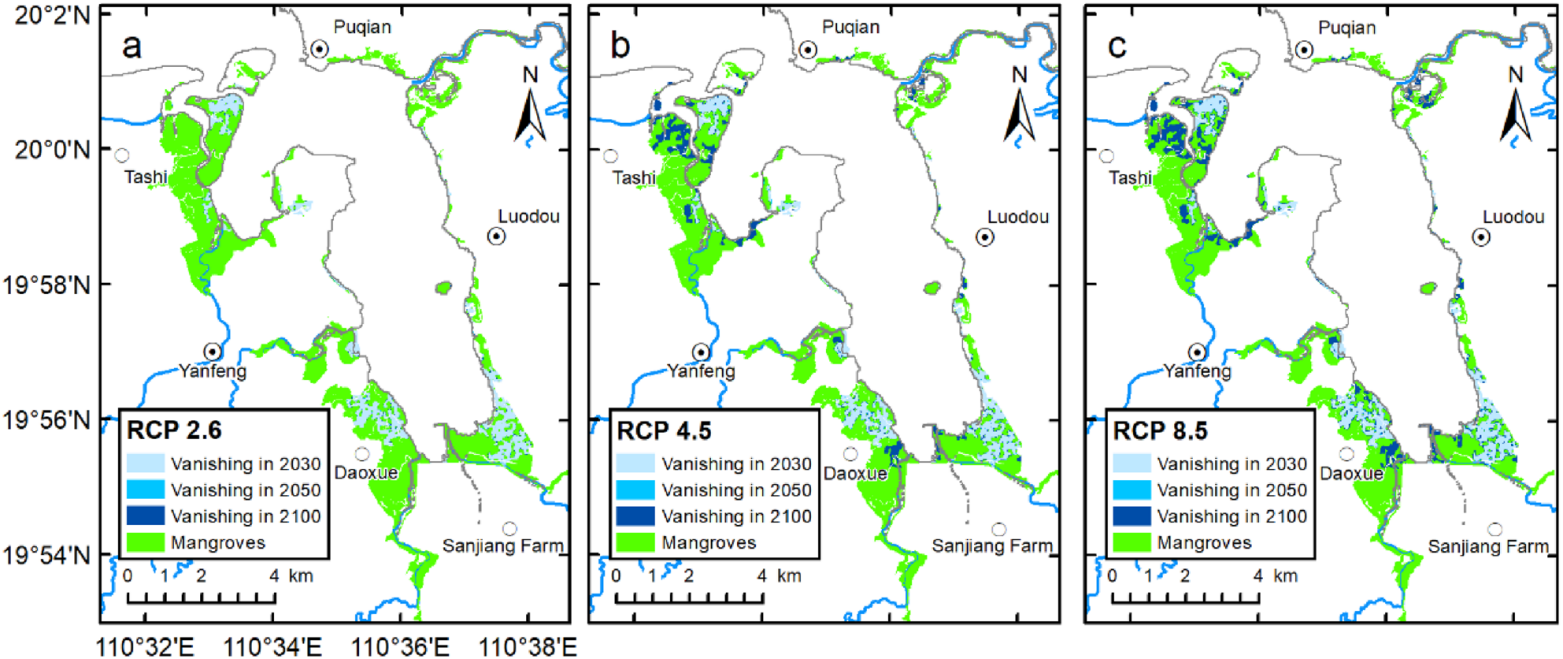
Potential loss of mangrove forests in Dongzhaigang under different climate scenarios (RCPs 2.6, 4.5, and 8.5). Maps generated in ArcMap v10.0 (https://www.esri.com/en-us/home).

Based on the predicted future rates of SLR under RCPs 2.6, 4.5, and 8.5 and on the vertical sediment accretion rates of Dongzhaigang mangrove wetlands, the mangroves are likely to be affected by rising sea levels by 2030, 2050, and 2100, respectively (Table 2, Fig. 6). Under the low GHG emission scenario (RCP 2.6), the area of the Dongzhaigang mangrove forest will only experience a small reduction: 16.40% (1.20–16.95%, likely range), 302 hm^2^ (22–312 hm^2^, likely range); 16.73% (1.20–17.82%, likely range), 308 hm^2^ (22–328 hm^2^, likely range); and 17.60% (1.14–31.02%, likely range), 324 hm^2^ (21–571 hm^2^, likely range) by 2030, 2050, and 2100, respectively (Table 2, Fig. 6a). This is because the vertical sediment accretion rate of Dongzhaigang mangrove will remain largely constant with increasing RSLR rate. Moreover, it should be noted that compared with 2030, the increase areas of mangroves inundation caused by SLR will be small by 2050 under three RCPs scenarios (Table 2). In contrast, under the intermediate and very high GHG emission scenarios (RCPs 4.5 and 8.5), Dongzhaigang mangrove is expected to be more significantly affected by SLR. Under RCP 4.5, 26.56% (16.19–40.74%, likely range) or 489 hm^2^ (298–750 hm^2^, likely range) of mangrove forest will likely be lost by the end of the century (Table 3, Fig. 6b). Under RCP 8.5, it is projected that 31.99% (18.14–50.73%, likely range) or 589 hm^2^ (334–934 hm^2^, likely range) of mangrove forest will be lost by 2100 (Table 2, Fig. 6c). Therefore, under RCPs 4.5 and 8.5, the impact of SLR on mangrove wetlands by 2100 is much higher than that of RCP 2.6, and is likely to result in > 26% of mangroves being lost, whereas under RCP 2.6, only 17% of mangroves are likely to be lost.

**Table 2:** Area (hm^2^) and percentage of future mangrove loss in Dongzhaigang under different climate scenarios (RCPs 2.6, 4.5, and 8.5) (likely ranges).

Year	RCP 2.6		RCP 4.5		RCP 8.5	
	Mean (hm^2^)	17–83% (likely)	Mean (hm^2^)	17–83% (likely)	Mean (hm^2^)	17–83% (likely)
2030	302 (22–312)	16.40% (1.20–16.95%)	302 (22–312)	16.40% (1.20–16.95%)	302 (22–314)	16.40% (1.20–17.06%)
2050	308 (22–328)	16.73% (1.20–17.82%)	312 (22–330)	16.95% (1.20–17.93%)	317 (298–335)	17.22% (16.19–18.20%)
2100	324 (21–571)	17.60% (1.14–31.02%)	489 (298–750)	26.56% (16.19–40.74%)	589 (334–934)	31.99% (18.14–50.73%)

**Table 3:** Core stations and depths.

Station	Location	Latitude	Longitude	Depth
HG	Hegang village	19.98°N	110.55°E	67 cm
SJ	Sanjiang farm	19.92°N	110.62°E	75 cm

Under RCP 2.6, the rate of RSLR around Dongzhaigang will reach 0.72 cm year^−1^ in 2030 and then decrease in 2050 and 2080 to 0.69 and 0.68 cm year^−1^, respectively (Table 1). However, under RCP 4.5 (8.5), by 2030, 2050, and 2100, the rate of RSLR will reach 0.72 (0.72), 0.73 (0.80), and 0.79 (10.1) cm year^−1^, respectively. By 2100, some mangroves in the northern part of Tashi village, the eastern part of Yanfeng, the northern part of Daoxue Village, and the northeastern part of the Sanjiang farm will likely be lost owing to SLR, and other coastal wetlands will also be impacted. Since the rate of RSLR around Dongzhaigang is higher than the global average survival threshold for mangroves (i.e., the SLR rate exceeds 7.0 mm year^−1^), the Dongzhaigang mangrove will be significantly affected by SLR, with a potential loss of 31–32%; however, the survival threshold will not increase (Table 2, Fig. 6).

## Discussion

### Reasons for historical changes in area of Dongzhaigang mangrove

Before the 1960s, Dongzhaigang mangrove was less disturbed by human activities and mainly evolved naturally, demonstrating seaward expansion. Between 1960 and the late 1980s, the area of the natural mangrove forests declined by nearly half (Fig. 2) because of the impact of human activities such as the use of mangrove land for planting trees and the reclamation of fish ponds^17,19,20,30,37^. After the establishment of the nature reserve in the 1990s, Dongzhaigang mangrove was still damaged or impacted by human activities such as shrimp farming and tourism or diseases such as outbreaks of *Sphaeromatidae*^18–20,31,33,38^. With the damage reducing to some extent during this period, the area of Dongzhaigang mangrove remained relatively stable thereafter (Fig. 2) because of the increased emphasis on protecting the mangrove ecosystem over the past three decades. Mangroves in a total area of 173 hm^2^ were planted in the Dongzhaigang reserve between 1980 and 1990, and an area of approximately 100 hm^2^ was preserved and kept alive^39^. Most mangrove areas located on China’s coast have experienced a similar change with loss first and recovery later in the past decades^40^. However, with the warming climate, there is growing concern that Dongzhaigang mangrove will be greatly affected by the ongoing SLR under the intermediate and very high GHG emission scenarios (RCPs 4.5 and 8.5), especially the mangroves in the northern part of Tashi village, the eastern part of Yanfeng, the northern part of Daoxue Village, and the northeastern part of Sanjiang farm (see Fig. 6). In other words, under RCP 4.5 (8.5) scenario, future RSLR will have a relatively large impact on the mangroves. Therefore, it is very important to discuss the impact of SLR on mangroves and improve their adaptive capacity.

**Figure 7:**
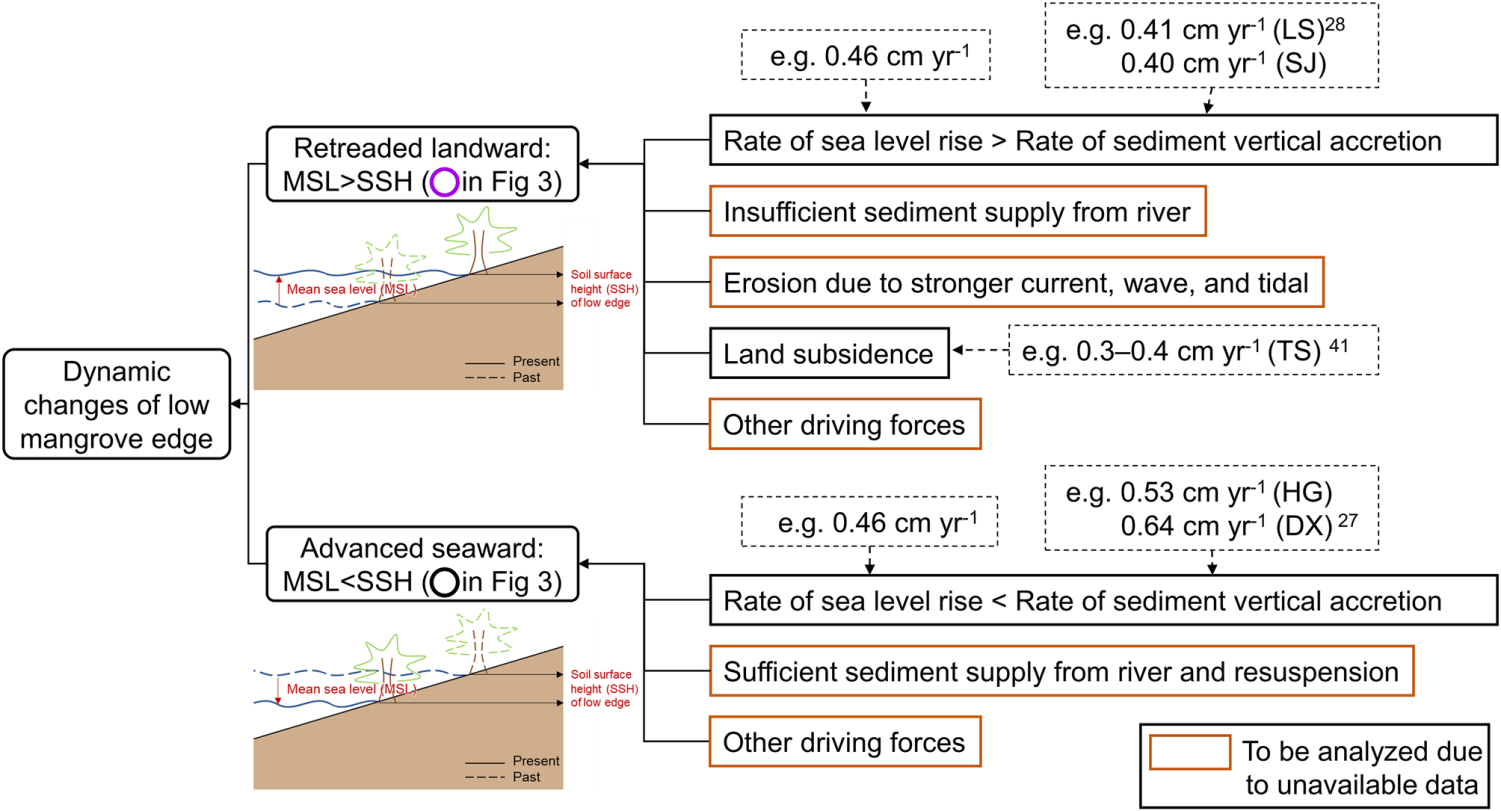
Conceptual framework diagram for the impact mechanism of SLR and other non-human forces on the mangrove wetland migrated seaward or landward. Figure created by R.C., R.D., and C.W.

Considering whether mangrove wetlands can maintain their soil surface height (SSH) above the mean sea level is critical for mangroves to adapt to SLR and survive. The changes in mangrove SSH generally depends on land subsidence and sediment accretion. Among these, sediment accretion can be affected by abiotic and biotic factors such as sediment supplies from the river transport, current, tidal and wave, mangrove root capture, and leaf litter accumulation. Due to limited available material, we mainly considered the dynamic changes in low mangrove edges in Dongzhaigang (Fig. 3) to analyze and illustrate the impact mechanism of SLR and other non-human directly driving forces on mangroves. The low mangrove edge migrated seaward or landward in Dongzhaigang owing to SLR and other natural driving forces, as illustrated in Fig. 7. The landward retreated or seaward advanced edges reflect the combined influence of SLR, sediment accretion, and land subsidence on the Dongzhaigang mangrove wetland. For example, the rate of land subsidence in northwest coast including Tashi district reached 0.3–0.4 cm year^−1^ in the last 50 years^41^. Ding et al.^42^ indicated that sedimentary deposition in the west coast of Dongzhaigang (including Tashi) has almost stopped since the Holocene. Moreover, Tan and Zhang^43^ revealed that the amount of sediment deposited annually to the Tashi bay in Dongzhaigang is only 1300 tons. In addition, the rate of sediment accretion in Sanjiang is 0.40 cm year^−1^ (less than the SLR rate of 0.46 cm year^−1^), which is conducive to mangrove wetland erosion. The combined effects of these natural driving forces can exacerbate the impact of SLR on the mangroves in Sanjiang, Tashi, and Luodou districts. In contrast, there are two smaller shore lines (black circles) in the northern part of Yangfeng and Daxue districts, advancing seaward. This is because the SSH of the low mangrove edge increased faster than that of SLR. The changes in SSH are generally attributed to sediment accretion rate, which is attributed to the sufficient or insufficient sediment supply from river transport, mangrove leaf litter accumulation, and root capture. When the rate of SSH of low mangrove edge is smaller (greater) than that of SLR, then the mangrove wetland migrates landward (seaward), as shown in Fig. 7.

### Adaptation responses

**Figure 8:**
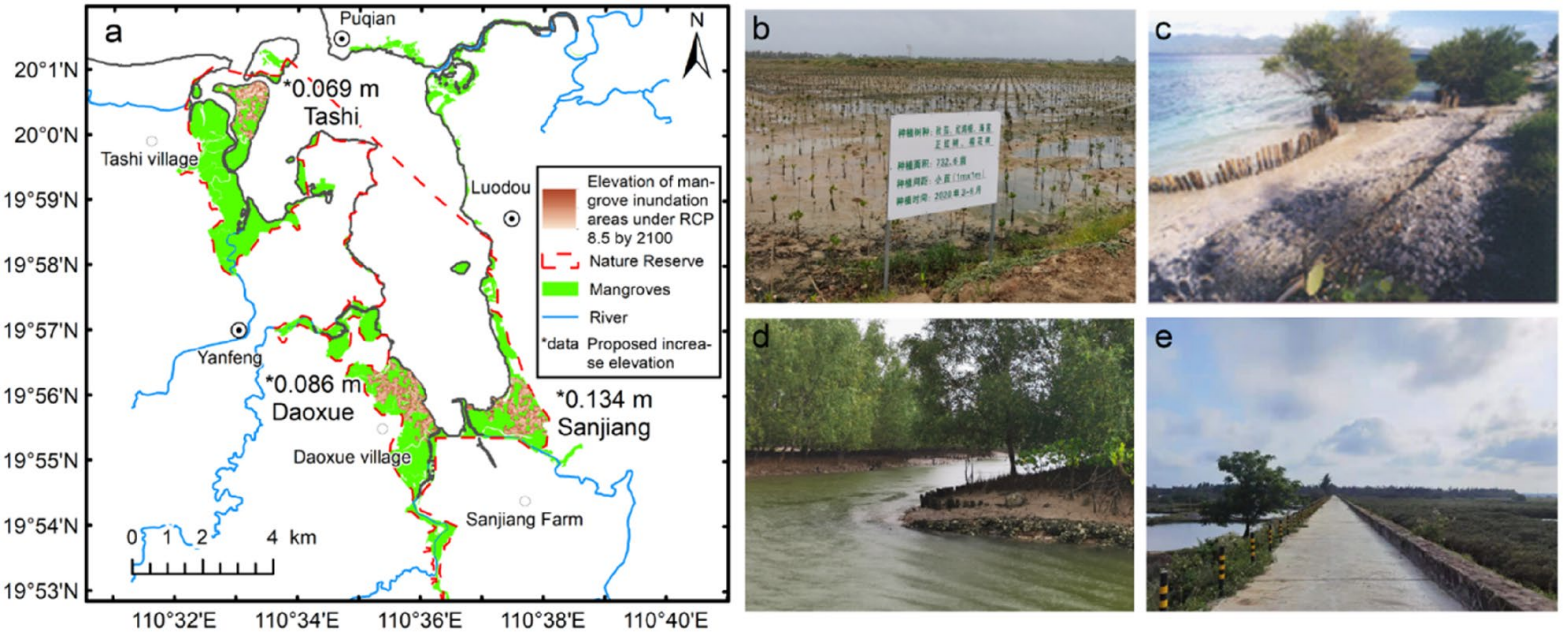
Measures for addressing SLR in coastal wetlands. (**a**) Heights of biological berms not less than 0.069, 0.086, and 0.134 m for the mangrove wetlands at the seaward side of Tashi village, Yanfeng, and Sanjian farm in Dongzhaigang, respectively; the shaded areas are projected to be inundated under RCP 8.5 by the end of 2100; (**b**) Restoration of mangrove from aquaculture and mudflat; (**c**,**d**) Biological embankment measures for sediment material acquisition in coastal wetlands (credit to: Poh Poh Wong); (**d**) Aquaculture ponds and cofferdams that block the mangrove naturally landward migrated. Maps generated in ArcMap v10.0 (https://www.esri.com/en-us/home).

In general, mangroves can adjust the soil surface elevation to adapt to SLR through landward migration. Adaptation measures such as sediment trapping, biological berm building, and restoration strategies are therefore suggested, as shown in Fig. 8a–d. In recent decades, as China has placed increasing emphasis on the protection of mangrove ecosystems, restoration techniques such as planting mangrove trees or reforestation have been implemented on the seaward side of the mangroves or in ponds on the landward side (Fig. 8b). Using biological berms such as wooden piles or oyster shells on the seaward side of the coast (Fig. 8c,d) can facilitate the mangrove wetlands to adjust soil surface elevation through sediment trapping and mitigate the coastal erosion and submergence due to SLR.

The analysis results showed that the northern part of Tashi, the eastern part of Yanfeng, the northern part of Daoxue, and the northeastern part of the Sanjiang farm in Dongzhaigang may be severely affected by SLR in the future. The northern part of Tashi is blocked by a tidal dyke at the rear; the southern parts of Tashi and Yanfeng are interspersed with dykes or cofferdams (Fig. 8e), village roads, and farming ponds at the rear (Fig. 8e); and Daoxue and the Sanjiang farm have farming ponds at the rear. Our investigation also indicated that approximately 80% of mangrove wetlands in China have tidal dykes or aquaculture ponds on the landward side^44^, as shown in Fig. 8e.

Based on the ecological restoration concept of “natural restoration as the mainstay and artificial intervention/support as a supplement,” the main restoration and protection measures that can be adopted for Dongzhaigang mangrove to mitigate the impact of SLR are listed below.

1. At the farming ponds on the landward side, in addition to the traditional model of returning the aquaculture ponds to forests for restoration in Tashi, Daoxue, and Sanjian (Fig. 8b), ecological aquaculture complexes (e.g., tile tank ecological farming in planted or naturally restored forest areas) can be built. This would not only restore natural hydrodynamic functions and improve water quality but also stabilize the livelihoods of fishermen and achieve sustainable development.
2. For the mangroves on the seaward side of Tashi village, Yanfeng, and the Sanjian farm in Dongzhaigang, the heights of biological berms (Fig. 8c,d) not less than 0.069, 0.086, and 0.134 m, shown in Fig. 8a, are recommended to be adopted for sediment material trapping to mitigate the impact of SLR by the end of 2100. Moreover, the tidal biological berms can facilitate an increase in the soil surface height of the low mangrove edges through sediment trapping, forming a habitat conducive to the growth of mangroves.
3. The amount of sediment such as silt and sand transported annually to Tashi bay in Dongzhaigang is only 1300 tons, which is much smaller than that of the river transport in the Jiulong river (2.23 million tons) in Xiamen, Fujian, and the Xijiang river (53.9 million tons) in Lingdingyang^43^, lowering the rate of vertical accretion of sediment. The inlet gates of the rivers of Dongzhaigang, such as the Tashi canal branch, and the cofferdams that block mangroves that are naturally landward migrated (Fi. 8e), can be removed and rebuilt or opened when needed. Adaptation measures such as returning the aquaculture ponds to forests for restoration, removing the cofferdams that block mangroves naturally landward migrated, and building biological berm for sediment trapping, would be helpful for enhancing the resilience of mangroves to SLR. This would also increase sediment transport, improve sediment trapping and siltation function of the mangrove wetlands, create a natural recovery environment for the mangrove growth, and enhance the adaptability of mangroves to SLR.
4. A comprehensive observation and monitoring system can be established. The application of remote sensing technology can be improved to understand the dynamic changes more accurately in mangroves. To monitor the changes in soil surface elevation of mangroves, a rod surface elevation table-marker horizon measurement system can be established in protected areas^13,45^ to further investigate the adaptive mechanisms of mangroves to SLR.

The characteristics of local habitats should also be considered in the conservation and restoration of mangroves. For example, in 1981, mangroves with areas of approximately 3 hm^2^ such as *Kandelia obovata* and *Bruguiera gymnorhiza* (L.) Lam. were planted on the bare beach west of the Sanjiang gate, but only 0.27 hm^2^ of *Kandelia obovata* survived owing to prolonged flooding. Mangroves such as *Kandelia obovata* and *Bruguiera sexangula* (Lour.) Poir. with areas of approximately 1 hm^2^ were planted on the bare beach at Tiaoyidu, but all of them died because of the hard substrate and high-salinity seawater^39^. Therefore, when adopting restoration measures such as mangrove planting, the salinity of seawater in the planted area and the nature of the soil, tides, and currents should be considered. Moreover, different mangrove species should be selected in different spatial zones, e.g., selecting resistant pioneer species, such as *Avicennia marina* (Forsk.) Vierh. and *Aegiceras corniculatum* (Linn.) Blanco., in the pioneer plant zone. In addition, the relationship between vegetation growth and spatial distribution in different growth periods (e.g., the width, density, and area of the forest) should be considered^46^. To improve the survival rate of pre-dyke afforestation plants, coastal engineering methods or beach herbaceous plants can be used beforehand^47^.

## Materials and methods

### Study sites

The Dongzhaigang mangrove is located at 19° 51′–20° 01′ N and 110° 32′–110° 37′ E. Dongzhaigang is a drowning valley bay formed by subsidence following the 1605 Qiongzhou Earthquake^28,48^. Nearly 700 million m^3^ year^−1^ of water flows into Dongzhaigang from rivers of Yanzhou, Luoya, Yanfeng East, and Yanfeng West. The rivers supply a large amount of sediment and form a wide mudflat, marshes, and wetlands for the growth of mangroves^19^. The Dongzhaigang Bay has a total area of 5240 hm^2^^49^ and a coastline length of approximately 80–84 km^50,51^. The soil type of Dongzhaigang mangrove is basalt, typical lateritic red soil, which is saline sandy loam or saline marsh soil with an approximate thickness of 1.0–1.5 m^52^.

## Materials

### Mangrove data

Three types of mangrove data were used in this study: (1) Historical sediment accretion rate data were collected from 1992 to 1994 at Linshi and Daoxue villages in Dongzhaigang mangrove with respective station codes of LS and DX^27,28^ (Fig. 1). (2) Supplementary investigations of sediment cores were carried out on the mangrove wetlands in Hegang village and Sanjiang farm. Peat drilling was performed to obtain four parallel, column-like sediment cores in December 2020 at an elevation of approximately 0.5–1.0 m above mean sea level^53^. The station codes were HG and SJ (Table 3, Fig. 1). (3) Mangrove map of China 2018 was derived from 2 m resolution satellite observations and field data^21^. Zhang et al.^54^ employed a mixed method of object-based image analysis, interpreter editing, and field surveys to analyze the 2 m resolution Gaofen-1 and Ziyuan-3 satellite images together with field data. The accuracy assessment of the confusion matrix showed that the Kappa coefficient reached 0.98, suggesting that the mangrove dataset had a high degree of thematic accuracy^54^. (4) Mangrove maps of China from 1986 to 2020 were derived from 30 m resolution Landsat 5, Landsat 7, and Landsat 8 Surface Reflectance Tier 1 satellite images and field data, and generated via U-net structure deep learning method.

### Sea level, elevation, and coastline data

The observed data from the Sea Level Bulletin of China 2020^22^ and the model data from the CMIP5 of the Intergovernmental Panel on Climate Change (IPCC) were obtained from Kopp et al.^24^.

The following datasets were used for elevation and coastline data: (1) Shuttle Radar Topography Mission (SRTM) digital elevation model (DEM) v3 with a spatial resolution of 30 m (National Aeronautics and Space Administration and National Geospatial-Intelligence Agency, https://earthdata.nasa.gov/), and (2) the Landsat 8 Operational Land Imager (OLI) with 30 m spatial resolutions (United States Geological Survey, https://earthexplorer.usgs.gov/).

## Methods

The area of Dongzhaigang mangrove was calculated as follows. Using the 2 m resolution remote sensing data of the spatial distribution of mangroves in China, the area of the mangrove was extracted from the national mangrove distribution data using ArcGIS after projection transformation. To determine the shoreline of Dongzhaigang, after radiometric calibration and atmospheric correction of the Landsat 8 OLI data, the multi-band spectral relationship method based on threshold methods^55^ (Eq. 1) was used to increase the number of water pixels. Threshold segmentation was then performed to obtain the shoreline data. Finally, visual interpretation was applied to exclude the boundary of independent water bodies such as ponds and lakes.

1 $${\text{Band}}3 + {\text{Band}}4 - {\text{Band}}5 - {\text{Band}}6 > {\text{T}},$$

where Band3 to Band6 correspond to the green, red, near-infrared, and short-wave infrared 1 bands in the Landsat 8 OLI data, respectively. T is the threshold, which depends on the extraction effect of the water body. In this study, the threshold was 500.

The sedimentation accretion rates in the mangrove were analyzed as follows. The constant flux-constant sedimentation rate model^56,57^ for ^210^Pb_ex_ (excess ^210^Pb) was used to estimate the rate of vertical accretion at sites HG and SJ. The constant sedimentation rate model is the most frequently used ^210^Pb dating model and is described in Eq. (2):

2 $$^{{{210}}} {\text{Pb}}_{{{\text{ex}}}} =^{{{210}}} {\text{Pb}} -^{{{226}}} {\text{Ra}} = \left( {^{{{210}}} {\text{Pb}}_{0} -^{{{226}}} {\text{Ra}}_{0} } \right)e^{{ - {\text{dl}}}} ,$$

where ^210^Pb–^226^Ra is the activity of ^210^Pb_ex_ in the sediment at depth l, ^210^Pb_0_–^226^Ra_0_ is the activity of ^210^Pb_ex_ in the initial surface sediment, and d is a constant obtained by fitting the experimental data. The deposition rate was calculated as V = λ/d, where λ is the decay constant of ^210^Pb (0.03 year^−1^).

To obtain the projected values and rates of SLR in the Dongzhaigang mangrove wetlands, the IPCC-CMIP5 multi-model data^24^ for the Haikou area in 2030, 2050, and 2100 under RCPs 2.6, 4.5, and 8.5 were calculated.

To analyze the impact of SLR on Dongzhaigang mangrove, two views of the SRTM DEM v3 data, named N20E110 and N19E110, were used to mosaic and extract the data for the study area. Global Mapper was applied to generate contours of the mangrove distribution area using the SRTM DEM data. The outer boundary of the mangrove (seaward boundary elevation) was at the mean sea level or slightly above, and the inner boundary (or maximum elevation within the forest) was at the mean high water spring level^50^. The contour closest to the outer boundary of the mangrove was selected, and its height was considered the current mean sea level height (H_0_).

Equation (3) was used to predict future changes in sea level:

3 $${\text{H}} = {\text{H}}_{0} - \Delta {\text{H}}_{{2000{-}2018}} + \Delta {\text{H}}_{{2000{-}{\text{N}}}} - {\text{V}}_{{\text{a}}} \times {\text{T}},$$

where H is the mean sea level height in Dongzhaigang in a future year. ΔH_2000–2018_ is the mean sea level change in Dongzhaigang from 2000 to 2018. ΔH_2000–N_ is the SLR in a future year (2030, 2050, or 2100) under different climate scenarios using 2000 as the base year for the CMIP5 data starting in 2000^24^. V_a_ is the rate of vertical sediment accretion. Here, we assumed that the vertical accretion rate will remain constant over the next hundred years. T is the time span.

The contour line corresponding to the future sea level calculated using Eq. (3) can be regarded as the outer boundary of mangroves in the future. Superimposed on a map of the mangrove forest, the area of future mangrove inundation can be calculated based on the following assumption: the area of future mangroves will be inundated if the vertical sediment accretion rates of the mangrove wetlands are lower than the rates of relative SLR in the future.

## Data Availability

The data presented in this study are available on reasonable request from the corresponding author.
