# Author Correction: Adaptive response of Dongzhaigang mangrove in China to future sea level rise

**DOI:** 10.1038/s41598-022-19680-w

**Published:** 2022-09-15

**Authors:** Rongshuo Cai, Ruyi Ding, Xiuhua Yan, Cuihua Li, Jiang Sun, Hongjian Tan, Wu Men, Haixia Guo, Cui Wang

**Affiliations:** 1 Third Institute of Oceanography, Ministry of Natural Resources, Xiamen, 361005 China; 2 Institute of Marine Sciences, Shantou University, Shantou, 515063 China; 3 School of Marine Sciences, Nanjing University of Information Science and Technology, Nanjing, 210044 China

Correction to: *Scientific Reports* 10.1038/s41598-022-15774-7, published online 7 July 2022

The original version of this Article contained errors in Figure 3 where the years "1986", "2000" and "2020" were incorrectly given as "2030", "2050" and "2100" in panels b, c, d, e, f, g, h, i and j. The original Figure 3 and accompanying legend appear below.

**Figure 3:**
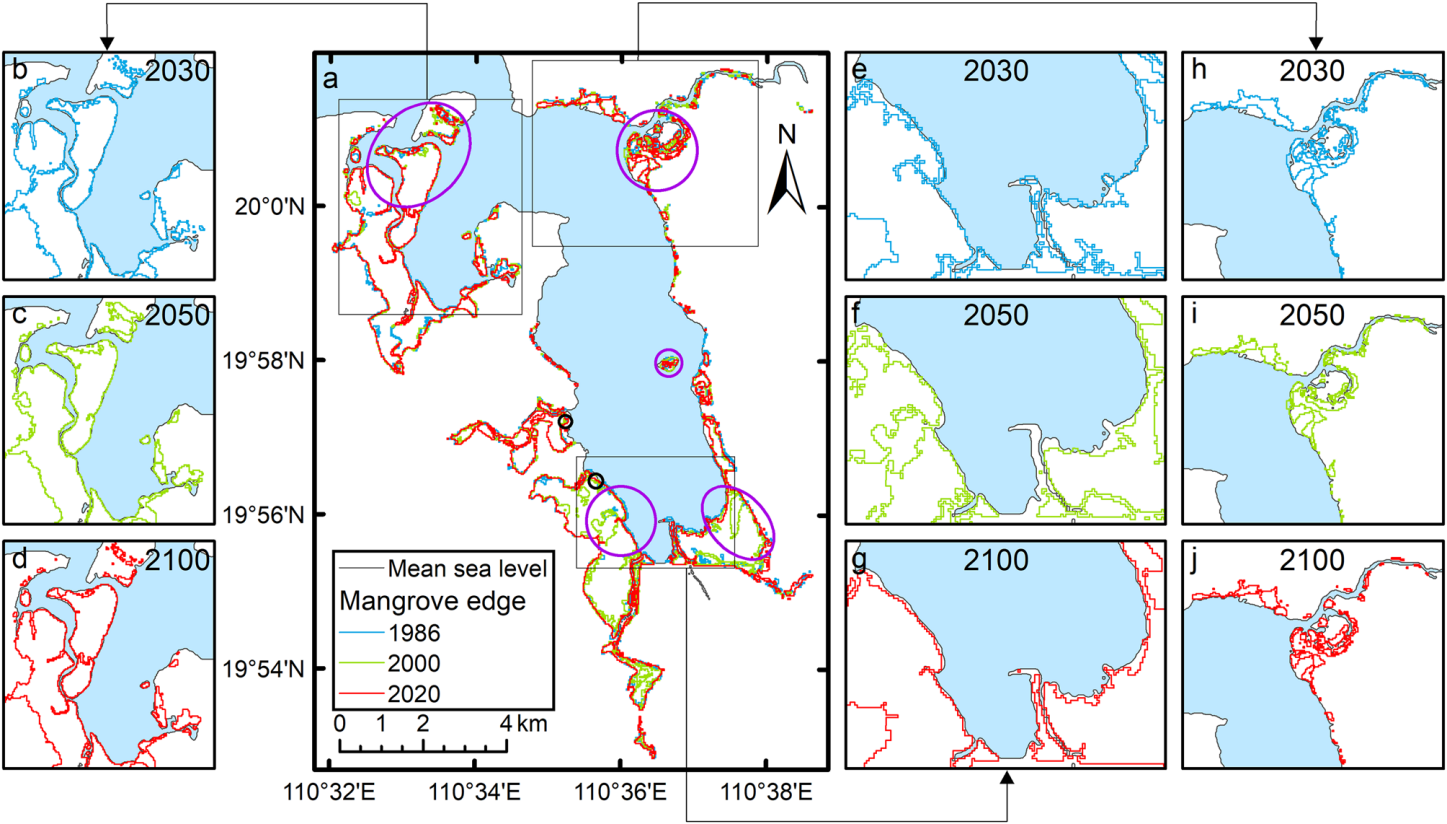
The dynamic changes in low mangrove edges in Dongzhaigang from 1986 to 2020. Maps generated in ArcMap v10.0 (https://www.esri.com/en-us/home).

The original article has been corrected.

